# Pepsin Hydrolysis of Orange By-Products for the Production of Bioactive Peptides with Gastrointestinal Resistant Properties

**DOI:** 10.3390/foods10030679

**Published:** 2021-03-23

**Authors:** Seyadeh Narges Mazloomi, Alireza Sadeghi Mahoonak, Leticia Mora, Mohammad Ghorbani, Gholamreza Houshmand, Fidel Toldrá

**Affiliations:** 1 Instituto de Agroquímica y Tecnología de Alimentos (CSIC), Avenue Agustín Escardino 7, Paterna, 46980 Valencia, Spain; samira.mazloomi@yahoo.com (S.N.M.); ftoldra@iata.csic.es (F.T.); 2 Department of Food Science & Technology, Gorgan University of Agricultural Sciences & Natural Resources, Gorgan 4918943464, Iran; moghorbani@yahoo.com; 3 The Health of Plant and Livestock Products Research Center, Department of Pharmacology, Mazandaran University of Medical Sciences, Sari 48175866, Iran; dr.houshmand_pharmaco@yahoo.com

**Keywords:** antidiabetic, ACEI inhibitory, antioxidant, peptide, hydrolysis

## Abstract

Recently, the use of bioactive compounds in improving human health has received more attention. The aim of the present study was to hydrolyze orange seed proteins using pepsin enzyme to obtain bioactive peptides as well as to study the stability of such activity after simulated gastrointestinal digestion conditions. The method was optimized using different enzyme concentrations from 1% to 3%, hydrolysis times between 2 and 5 h, and an optimal temperature of 33 °C. Biological activities including α-glucosidase inhibition, α-amylase inhibition, Angiotensin I-Converting Enzyme (ACEI) inhibition, ferric reducing antioxidant power, and 2,2-diphenyl-1-picrylhydrazyl (DPPH) radical scavenging activity were evaluated. According to the results, a significant higher value of the biological activity (*p* < 0.05) was observed using an enzyme ratio of 0.03 E/S and hydrolysis time of 3.5 h. After size-exclusion chromatography separation, fractions 45–49 and 50–54 showed the highest biological roles such as antioxidant, ACEI inhibitory, and hypoglycemic. Fractions with the highest biological activity were purified using RP-HPLC and analyzed using nano-liquid chromatography and mass spectrometry. The results obtained after simulated gastrointestinal digestion indicated that peptide fractions obtained after chromatographic separation significantly maintain their activity.

## 1. Introduction

In recent years, the importance of using bioactive compounds in diet and their role in reducing diseases and disorders due to improper nutrition has increased. Proteins and peptides are the most important bioactive compounds among natural sources [1]. When proteins and peptides have been hydrolyzed, they can exert an important biological role via antioxidant [2,3,4,5,6], antihypertensive [4,6,7,8], antidiabetic [9,10], or anticancer [2] activities. Therefore, numerous studies have been focused on the production of bioactive peptides. These studies include the production of peptides from royal jelly [11], orange seed flour [12], pumpkin seed [13], mung bean [14], rainbow trout skin [15], whey [16], or tomato seed [17]. Protein hydrolysates obtained from food industry by-products can be used as a rich source of bioactive peptides in nutritional supplements.

The orange seed is an important by-product obtained from the juice production industry. The juice industry considers seed flour as a by-product that contains between 17.9% to 26.5% of protein [18]. In fact, the protein obtained from defatted flour can be used as a low-cost source for the generation of peptides through hydrolysis that could have an important role as antioxidant, anticancer, antidiabetic, or antihypertensive peptides.

The main objective of the present study was to hydrolyze orange seed proteins using the pepsin enzyme, a protease from porcine gastric mucosa, to obtain bioactive peptides showing the highest antioxidant, antihypertensive, and hypoglycemic capacity as well as to study the stability of such activity after simulated conditions of gastrointestinal digestion. After the separation of peptides using size-exclusion chromatography and RP-HPLC, the most active fractions were analyzed to identify the peptides using mass spectrometry in tandem before and after simulated gastrointestinal digestion.

## 2. Materials and Methods

### 2.1. Materials

Pepsin enzyme (protease from porcine gastric mucosa), porcine pancreatic α-amylase, porcine pancreatic lipase, trypsin (from hog pancreas), chymotrypsin (from bovine pancreas), porcine bile extract, and Angiotensin I-Converting Enzyme (ACEI) were obtained from Millipore-Sigma (St. Louis, MO, USA). NaOH, HCl, ethanol, sodium phosphate, potassium ferricyanide, trichloroacetic acid, 2,2-diphenyl-1-picrylhydrazyl (DPPH), ferric chloride, acarbose, and CaCl_2_, were purchased from Sharlau (Barcelona, Spain). All chemicals used in the study were of analytical grade.

### 2.2. Preparation of Orange Seed Protein Concentrates

The defatted orange seed flour (containing 22.5% protein) was mixed with bidistilled water (at a ratio of 1:20 at room temperature). Then, NaOH at a concentration of 1 N was used to get a pH of 10 and the mixture was homogenized during 1 h at room temperature and later centrifuged (15 min, 12,000 rpm) using an Avanti J-26S XP centrifuge (Beckman Coulter Inc., Indianapolis, IN, USA). Later, HCl at a concentration of 1 N was used to decrease the pH to 3 and centrifuge to obtain a clean pellet (15 min, 12,000 rpm), which was washed with bidistilled water and freeze dried (SCANVAC, Labogene ApS, Lillerød, Denmark) [19]. The protein concentrates contained 75.1% protein and were used for the hydrolysis process.

### 2.3. Enzymatic Hydrolysis Conditions

The hydrolysis condition was optimized using different enzyme concentrations from 0.01 to 0.03 E/S, hydrolysis times between 2 and 5 h, and the optimal temperature of 33 °C in a shaker incubator (Radleys, Essex, UK). The treatments used were T1: 0.01 E/S, 2 h; T2: 0.02 E/S, 2 h; T3: 0.03 E/S, 2 h; T4: 0.01 E/S, 3.5 h; T5: 0.02 E/S, 3.5 h; T6; 0.03 E/S, 3.5 h; T7: 0.01 E/S, 5 h; T8: 0.02 E/S, 5 h; T9: 0.03 E/S, 5 h (optimal temperature: 33 °C and protein concentration of 0.05% *w*/*v*). At the end of each treatment, the enzyme was inactivated at 85 °C for 15 min and then the suspension was centrifuged (Suprema 25, TOMY, Tagara, Nerima-ku, Japan) at 4 °C for 20 min at 12,000 rpm for deproteinization. Finally, the supernatant was collected, lyophilized, and kept at −20 °C until use. The bioactivity assays including DPPH scavenging activity; ferric reducing power; and ACEI, α-amylase, and α-glucosidase inhibitory activities were done for each sample, and those showing the highest activity value in all the tests were selected for the next steps [20]. For the preparation of the protein hydrolysate for identification purpose, the T6 treatment, which showed the best bioactivity results, was prepared at a concentration of 10% (*w*/*v*).

### 2.4. DPPH Radical Scavenging Activity Assay

In order to perform this assay, a total of 100 μL of protein hydrolysate at a concentration of 0.1 mg/mL was incubated with 500 μL of ethanol and 125 μL of 2,2-diphenyl-1-picrylhydrazyl (DPPH) at a concentration of (0.02%). After incubation for 60 min at room temperature, the absorbance of samples was read at 517 nm and DPPH radical scavenging activity (%) was calculated as [21]: (Absorbance negative control − Absorbance sample)/Absorbance negative control × 100. Bidistilled water and butilhidroxitolueno (E-321, BHT, 1 μg/μL) were used as negative and positive controls, respectively.

### 2.5. Ferric Reducing Antioxidant Power Assay

For this, 250 μL of protein hydrolysate (0.1 mg/mL) was added to 250 μL of 200 mM sodium phosphate buffer prepared at a pH of 6.6 together with 250 μL potassium ferricyanide at a concentration of 10 mg/mL. Then, samples were stirred for 20 min at a temperature of 50 °C. After that, 250 μL of trichloroacetic acid (100 mg/mL) was added to the solution and then centrifuged for 10 min at 1650 rpm. Then, 500 μL of supernatant, 500 μL distilled water, and 100 μL of ferric chloride (1 mg/mL) were mixed together, stored at room temperature for 10 min, and measured at 700 nm. Distilled water was employed as the negative control and BHT (1 μg/μL) as the positive control [22].

### 2.6. ACEI Inhibition Activity Assay

Briefly, 50 μL of protein hydrolysate (0.1 mg/mL) was added to 50 μL of Tris-base buffer (150 mM, pH 8.3) containing ACEI (3 mU/mL) together with 200 μL of 1.125 M NaCl and 10 mM Abz-Gly-Phe(NO_2_)-Pro prepared with the same Tris buffer. The reaction mixture was stored at 37 °C for 60 min [23]. ACEI-inhibitory activity (%) = ((Fluorescence t60 − Fluorescence t0)/(Fluorescence control t60 − Fluorescence control t0)) * 100. Bidistilled water and captopril (10 μM) were used as the negative and positive control, respectively.

### 2.7. α-Amylase Inhibition Activity Assay

The α-amylase inhibition activity was tested using a commercial kit (SPINREACT, ref amylase-LQ, GI, Spain). Briefly, 80 μL of protein hydrolysate (0.1 mg/mL) was mixed with 40 μL of α-amylase enzyme (from porcine pancreas, A3176, 1 μU). The mixture was stored for 10 min at 37 °C and 160 μL of the 2-chloro-4-nitrophenyl-α-D-maltotrioside (CNPG3) was added. The new mixture was stored at 37 °C for 5 min. The absorbance was measured at 405 nm. The α-amylase inhibition activity was calculated as follows: α-amylase inhibition activity (%) = (Absorbance negative control − Absorbance sample)/(Absorbance negative control) × 100. Phosphate buffer 20 mM (pH 6.8) and acarbose (2 mg/mL) (SPINREACT, ref Amylase-LQ, GI, Spain) were used as the negative and positive control, respectively.

### 2.8. α-Glucosidase Inhibition Activity Assay

This assay was carried out using a commercial kit (Sigma-Aldrich, α-glucosidase Activity Assay Kit, St. Louis, MO, USA). Thus, 20 μL of protein hydrolysate was added to 50 μL of 1 mg/mL of α-glucosidase enzyme from *Saccharomyces cerevisiae,* then the sample was stored at 37 °C for 5 min, and 200 μL of the Master Reaction Mix containing 200 μL of pH 7 buffer and 8 μL of α-NPG substrate were added and incubated again at 37 °C for 60 min. The absorbance was measured at 405 nm. α-glucosidase inhibition activity (%) = (Absorbance control − Absorbance sample)/(Absorbance control) × 100. Bidistilled water and acarbose were used as the negative and positive control, respectively.

### 2.9. Size-Exclusion Chromatography (SEC) Separation of Hydrolyzed Protein

For SEC separation, 5 g of hydrolyzed protein was mixed with 35 mL of HCl (0.01 N). Then, three volumes of ethanol were added to this solution and stored at 4 °C during the night for deproteinization. The mixture was centrifuged at 12,000 rpm for 20 min at 4 °C to remove the precipitated proteins, and ethanol was evaporated using a rotary evaporator. Samples were lyophilized overnight (SCANVAC, Labogene ApS, Denmark). Before injection into the SEC column, 1 g of the dried sample was resuspended in 10 mL of 0.01 N HCl and filtered through a 0.45 μm syringe filter to remove non-soluble impurities. A total of 5 mL of the sample was injected on the Sephadex G25 column (2.5 × 65 cm) (Amersham Biosciences, Uppsala, Sweden). The separation process was performed using a flow rate of 15 mL per hour of degassed 0.01 N HCl, and 5 mL peptide fractions were collected using an automatic collector. Finally, the absorbance was measured at 254 and 280 nm in a Cary 60 UV-Vis spectrophotometer (Agilent Technologies, Palo Alto, CA, USA), and the biological activities were also assayed in all fractions [24,25].

### 2.10. RP-HPLC Separation of SEC Fractions

The most active peptide fractions pooled using SEC were filtered and pulled together for the HPLC separation. Then, they were filtered using a 0.45 μm nylon membrane filter, and 30 μL of sample was injected in an 1100 HPLC instrument (Agilent Technologies, Palo Alto, CA, USA) using a Symmetry C18 column (4.6 × 250 mm, 5 μm) from Waters Co. (Milford, MA, USA). Two solvents were used in the analysis: (A) 0.1% TFA in bidistilled water and (B) 0.085% TFA (*v*/*v*) in acetonitrile and bidistilled water in a proportion of 60:40, *v*/*v*. A and B solvents were filtered using a 0.45 μm nylon membrane, and the potential air of the solvents was removed using ultrasonication. The main HPLC conditions applied in the separation of the peptides were as follows: 100% A for 2 min and a gradient to 50% B for 50 min. The separation was done at a flow rate of 1 mL/min, and absorbance was measured at 214 nm. Fractions of 1 mL were collected for further analysis of their bioactivity [24,25].

### 2.11. In Vitro Gastrointestinal Digestion

This method was designed in two phases separately to cover both gastric and intestinal conditions. The simulated digestion under gastric conditions was done using 2 mL of 0.01 N HCl at pH 3, where a total of 500 mg of the freeze-dried most active RP-HPLC fractions were added. Pepsin at a concentration of 2000 U/mL and CaCl_2_ were also added with the aim to get a final enzyme concentration of 0.075 mM, and samples were stored for 3 h at 37 °C. In order to carry out the simulated intestinal digestion, the pH was increased to 7 using 1 M NaOH. Then, porcine pancreatic α-amylase (200 U/mL), porcine pancreatic lipase (2000 U/mL), trypsin (from hog pancreas, 100 U/mL), chymotrypsin (from bovine pancreas, 25 U/mL), and porcine bile extract (10 mM) were added. Furthermore, CaCl_2_ to get a final concentration of 0.3 mM was included in the mix, and the sample was incubated for 2 h at 37 °C. To stop the hydrolysis, samples were heated at 95 °C for 2 min. Finally, samples were centrifuged 12,000× *g* at 4 °C for 10 min to remove the precipitated proteins, and the supernatant was lyophilized [26].

### 2.12. Identification of Peptides Using Mass Spectrometry in Tandem

The mass spectrometry in tandem analysis was done using an Eksigent Nano-LC Ultra 1D Plus system (AB Sciex, Redwood City, CA, USA) that was connected to a quadrupole-time-of-flight (Q ToF) TripleTOF^®^ 5600 and to a nanoelectrospray ionization source (AB Sciex Instruments, Framingham, MA, USA). The RP-HPLC fractions showing the highest biological activity were concentrated/cleaned with Zip Tip C-18 tips (Millipore, Billerica, MA, USA). The sample was eluted with 5 μL elution solution according to the manufacturer conditions and injected into the mass spectrometry in tandem (MS/MS) instrument. Samples were preconcentrated on an Eksigent C18 trap column (3 µm, 350 µm × 0.5 mm) (Eksigent of AB Sciex, Dublin, CA, USA), using TFA 0.1% *v*/*v* as the mobile phase. After 5 min of preconcentration, the trap column was automatically changed with a nano-HPLC capillary column (3 μm, 75 μm × 12.3 cm, C18) from Nikkyo Technos Co, Ltd. (Toshima City, Tokyo, Japan). Elution was carried out with a linear gradient of 5% to 35% acetonitrile in 0.1% formic acid for 90 min at a flow rate of 300 nL/min. The sample was ionized by applying 2.8 kV to the spray emitter. Analysis was carried out in data-dependent mode. Survey MS1 scans were acquired from 350 to 1250 *m*/*z* for 250 ms. The quadrupole resolution was set to “UNIT” for MS/MS experiments, which were acquired 100–1500 *m*/*z* for 50 ms in “high sensitivity” mode. The following switch criteria were used: charge: 1+ to 5+; minimum intensity; 70 counts per second (cps). Up to 25 ions were selected for fragmentation after each survey scan. Dynamic exclusion was set to 15 s. Automatic spectrometry and peak list generation was done using Mascot Distiller software (Matrix v2.4.2.0 Science, Inc., Boston, MA, USA). The identification of the sequences was done searching the obtained spectra peak list in the protein database, UniProt, and the Mascot Distiller software with the search engine Mascot interface software 2.2.

### 2.13. Statistical Analysis

Analysis of data was performed using SPSS software (v 19.0, SPSS Inc., Chicago, IL, USA). Each experiment was repeated in triplicate. To compare the mean values, the Duncan’s test was employed at 5% significance level. An Excel software (Microsoft Excel Worksheet (.xlsx, US, 2013) was also employed to prepare the figures.

## 3. Results

### 3.1. Evaluation of Biological Activity of the Hydrolyzed Proteins

#### 3.1.1. Antioxidant Activity

**Figure 1:**
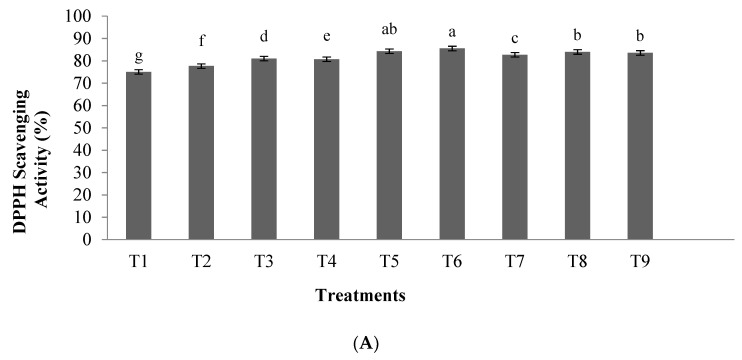

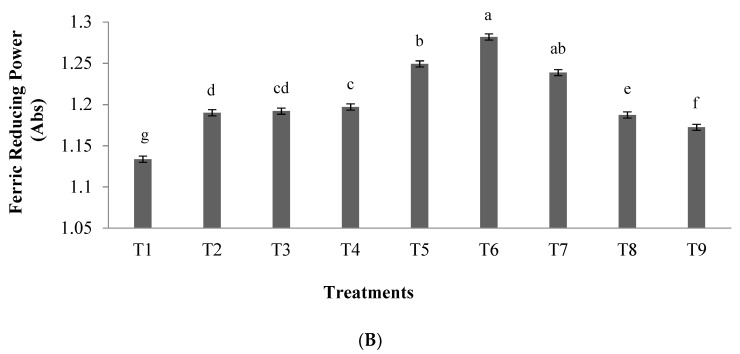
(**A**) 2,2-diphenyl-1-picrylhydrazyl (DPPH) scavenging activity of orange seed proteins hydrolyzed using pepsin enzyme. (**B**) Fe^3+^ reducing activity of orange seed proteins hydrolyzed using pepsin enzyme. Differences in letters indicate significant differences (*p* < 0.05) in the observed activity. Treatments: T1: 0.01 E/S, 2 h; T2: 0.02 E/S, 2 h; T3: 0.03 E/S, 2 h; T4: 0.01 E/S, 3.5 h;, T5: 0.02 E/S, 3.5 h; T6; 0.03 E/S, 3.5 h; T7: 0.01 E/S, 5 h;, T8: 0.02 E/S, 5 h; T9: 0.03 E/S, 5 h (temperature of 33 °C and protein concentration of 0.05% *w*/*v*).

The results of the DPPH scavenging activity of hydrolyzed orange seed proteins prepared using pepsin is shown in Figure 1A, whereas the results of ferric reducing activity are illustrated in Figure 1B. Our results revealed that the enzyme to substrate ratio and hydrolysis time had significant effects on DPPH scavenging activity and ferric reducing activity in orange seed protein hydrolysates (*p* < 0.05). The highest antioxidant activity was observed in the 0.03 E/S ratio and hydrolysis time of 3.5 h (T6), which showed radical DPPH inhibitory activity and ferric reducing capacity of 85.53% and 1.282, respectively. BHT (1 μg/μL) was used as a positive control and showed a radical DPPH inhibitory activity and ferric reducing capacity of 89.6% and 2.13, respectively.

#### 3.1.2. ACEI-Inhibitory Activity

**Figure 2:**
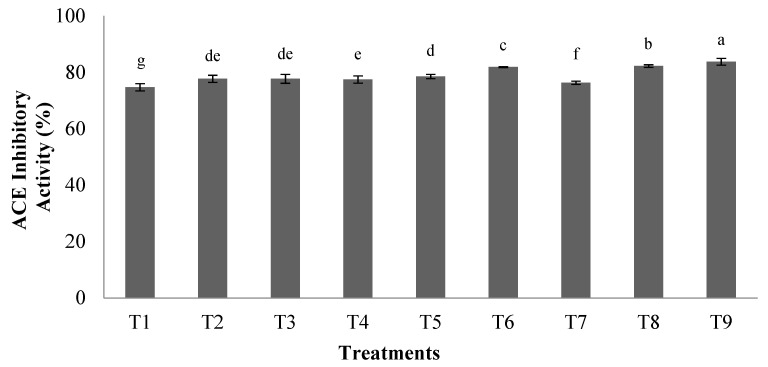
Angiotensin I-Converting Enzyme (ACEI)-inhibitory activity of hydrolyzed orange seed proteins hydrolyzed using pepsin enzyme. Differences in letters indicate significant differences (*p* < 0.05) in the observed activity. Treatments: T1: 0.01 E/S, 2 h; T2: 0.02 E/S, 2 h; T3: 0.03 E/S, 2 h; T4: 0.01 E/S, 3.5 h; T5: 0.02 E/S, 3.5 h; T6; 0.03 E/S, 3.5 h; T7: 0.01 E/S, 5 h; T8: 0.02 E/S, 5 h; T9: 0.03 E/S, 5 h (temperature of 33 °C and protein concentration of 0.05% *w*/*v*).

The ACEI-inhibitory activity of orange seed protein hydrolysates prepared using the pepsin enzyme at different conditions is presented in Figure 2. Increasing hydrolysis time and ratio of enzyme/substrate led to an increase in the inhibitory activity of ACEI enzyme in hydrolyzed protein samples. The highest ACEI-inhibitory activity (83.70%) was obtained at an enzyme to substrate ratio of 0.03 E/S and hydrolysis time of 5 h (T9) (*p* < 0.05). Captopril (10 µM) was also used as a positive control and showed ACEI-inhibitory activity of 100%.

#### 3.1.3. α-Amylase and α-Glucosidase Inhibitory Activity

**Figure 3:**
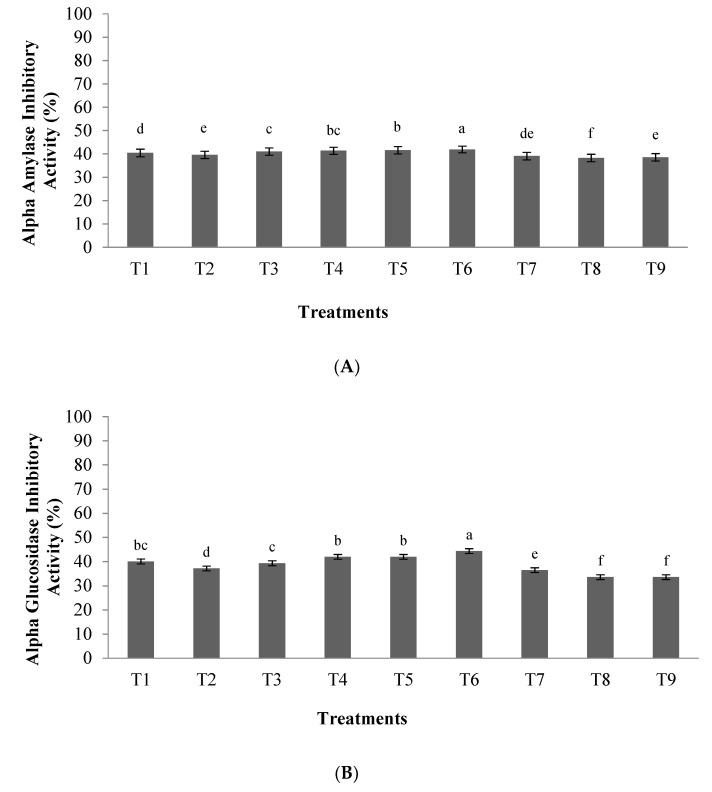
(**A**) α-amylase inhibitory activity of orange seed proteins hydrolyzed using pepsin enzyme, (**B**) α-glucosidase inhibitory activity of orange seed proteins hydrolyzed using pepsin enzyme. Differences in letters indicate significant differences (*p* < 0.05) in the observed activity. Treatments: T1: 0.01 E/S, 2 h; T2: 0.02 E/S, 2 h; T3: 0.03 E/S, 2 h; T4: 0.01 E/S, 3.5 h; T5: 0.02 E/S, 3.5 h; T6; 0.03 E/S, 3.5 h; T7: 0.01 E/S, 5 h; T8: 0.02 E/S, 5 h; T9: 0.03 E/S, 5 h (temperature of 33 °C and protein concentration of 0.05% *w*/*v*).

The antidiabetic potential of orange seed proteins hydrolyzed using the pepsin enzyme was evaluated based on the inhibitory activity of α-amylase and α-glucosidase enzymes, and their outcomes are reported in Figure 3A,B, respectively. Our results showed that α-amylase and α-glucosidase inhibitory activities of hydrolyzed orange seed proteins were affected by the ratio of enzyme to substrate and hydrolysis time (*p* < 0.05). According to the results, the highest antidiabetic potential (42.35% for α-amylase inhibitory activity and 45.39% for α-glucosidase inhibitory activity) was observed in the enzyme to substrate ratio of 0.03 E/S ratio and hydrolysis time of 3.5 h (T6) (*p* < 0.05). Acarbose (2 mg/mL) was also used as a positive control and showed α-amylase and α-glucosidase inhibitory activities of 80.12% and 86.42%, respectively.

### 3.2. Fractionation of Hydrolyzed Orange Seed Proteins Using SEC

**Figure 4:**
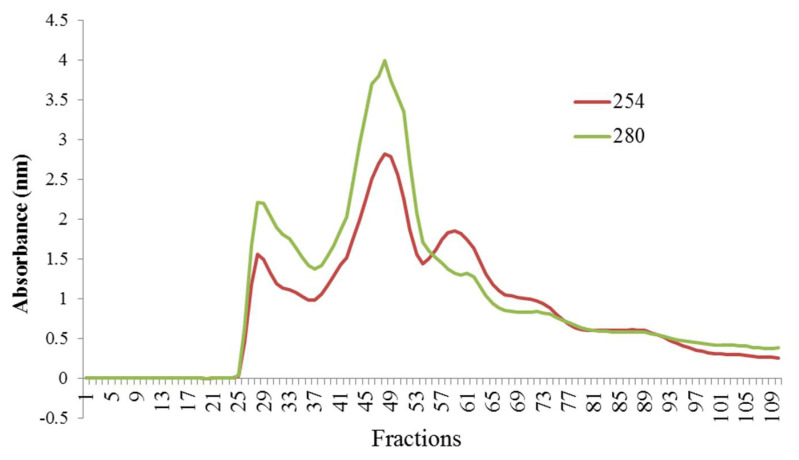
Size-exclusion chromatography (SEC) profile of orange seed proteins (T6; 0.03 E/S, 3.5 h) hydrolyzed using pepsin enzyme.

In order to obtain a more precise understanding about the effective factors affecting the antioxidant, antihypertensive, and antidiabetic activities of hydrolyzed orange seed proteins, the samples (T6) were fractionated using SEC. The absorbance of the fractions was measured at 254 and 280 nm. The results are shown in Figure 4, where the hydrolysis products are divided into three main absorbance peaks.

These peaks were ranged from 200 to 70,000 Da according to the results obtained after the analysis of standard proteins. The first peak corresponds to molecular weight from 13,000 to 70,000 Da, the second peak from 1400 to 13,000 Da. The last peak was related to low-molecular-weight components, as they eluted later than bacitracin standard (1400 Da).

### 3.3. Evaluation of Biological Activity in Peptides Isolated from SEC

**Figure 5:**
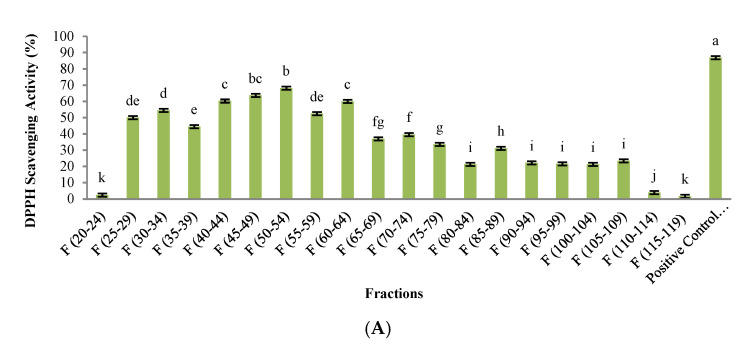

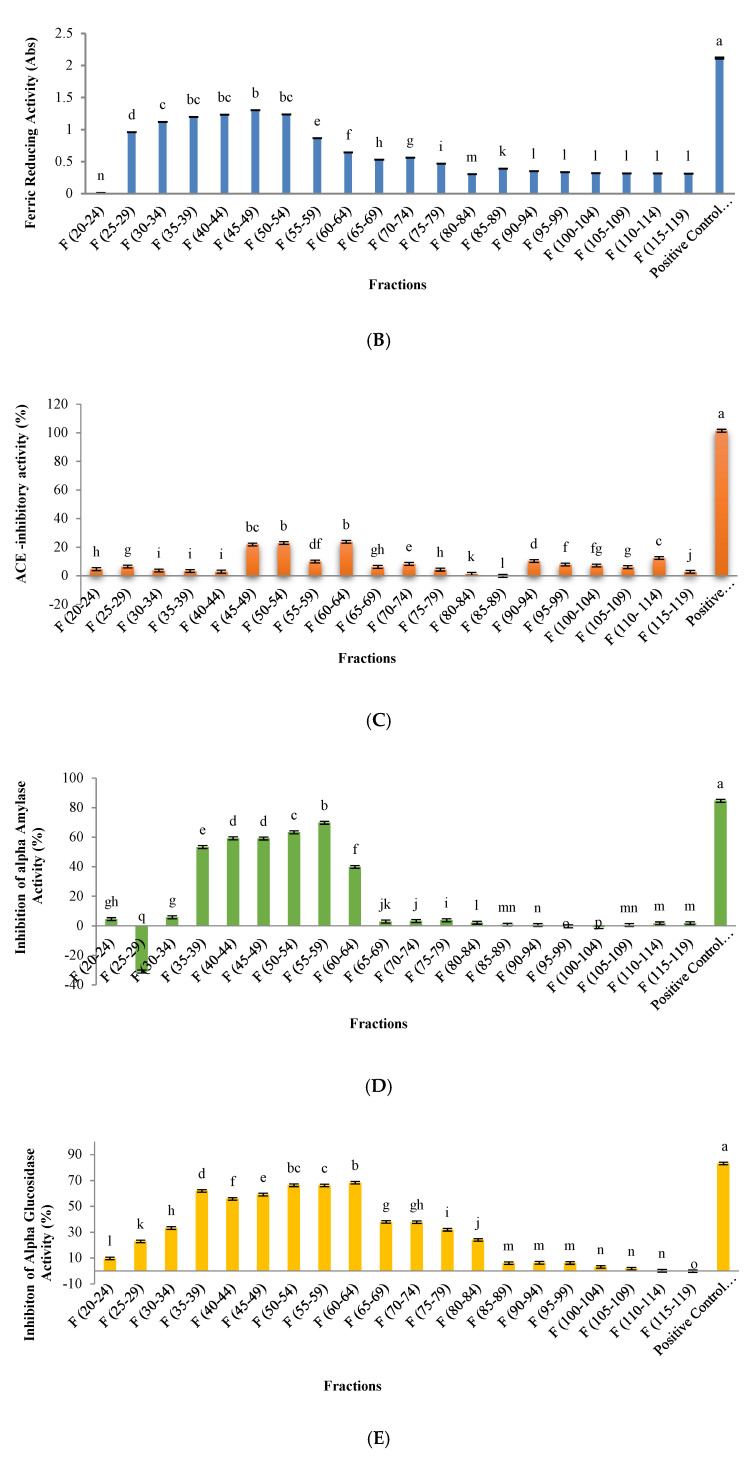
DPPH scavenging activity (**A**), ferric reducing capacity (**B**), ACEI-inhibitory activity (**C**), α-amylase inhibitory activity (**D**), and α-glucosidase inhibitory activity (**E**) of hydrolyzed orange seed peptides fractions 49–45 and 54–50 isolated from SEC. Differences in letters indicate significant differences (*p* < 0.05) in the observed activity. The last bar corresponds to the positive control used in each assay.

The results of DPPH scavenging, ferric reducing, ACEI-inhibitory, α-amylase inhibitory, and α-glucosidase inhibitory activities of peptide fractions isolated from SEC are presented in Figure 5.

Our observations indicate that the maximum levels of DPPH scavenging activity and the ferric reducing power of pepsin were detected in 45–49 and 50–54 peptide fractions. The DPPH scavenging activity values were 63.58% and 68.10%, and the ferric reducing power capacity was 1.301 and 1.236, respectively. Moreover, the highest ACEI-inhibitory activity of 87.22% was observed in peptide fractions 50–54 with molecular weight less than 13,000 Da. Additionally, fractions 60–64 showed good ACEI-inhibitory activity. On the other hand, the maximum inhibitory activity of α-amylase (77.8%) was found in peptide fractions 55–59 with molecular weights less than 13,000 Da although high inhibitory values were also described from fraction 35 to fraction 64. Regarding α-glucosidase inhibitory activity, the highest value was observed for fractions 50–54, 55–59, and 60–64; although very good values of inhibitory activity were observed from fraction 35 to fraction 64 (Figure 5E).

Finally, the fractions 44–49 and 50–54 were selected for further separation analysis as both of them showed good results in the assayed biological activities, so potential multifunctional peptides could be present in those fractions. Thus, they were pulled together and further analyzed using RP-HPLC.

### 3.4. Purification and Isolation of Peptide Fractions Using RP-HPLC

**Figure 6:**
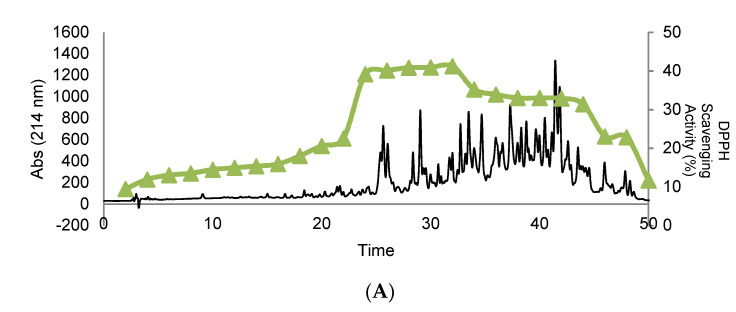

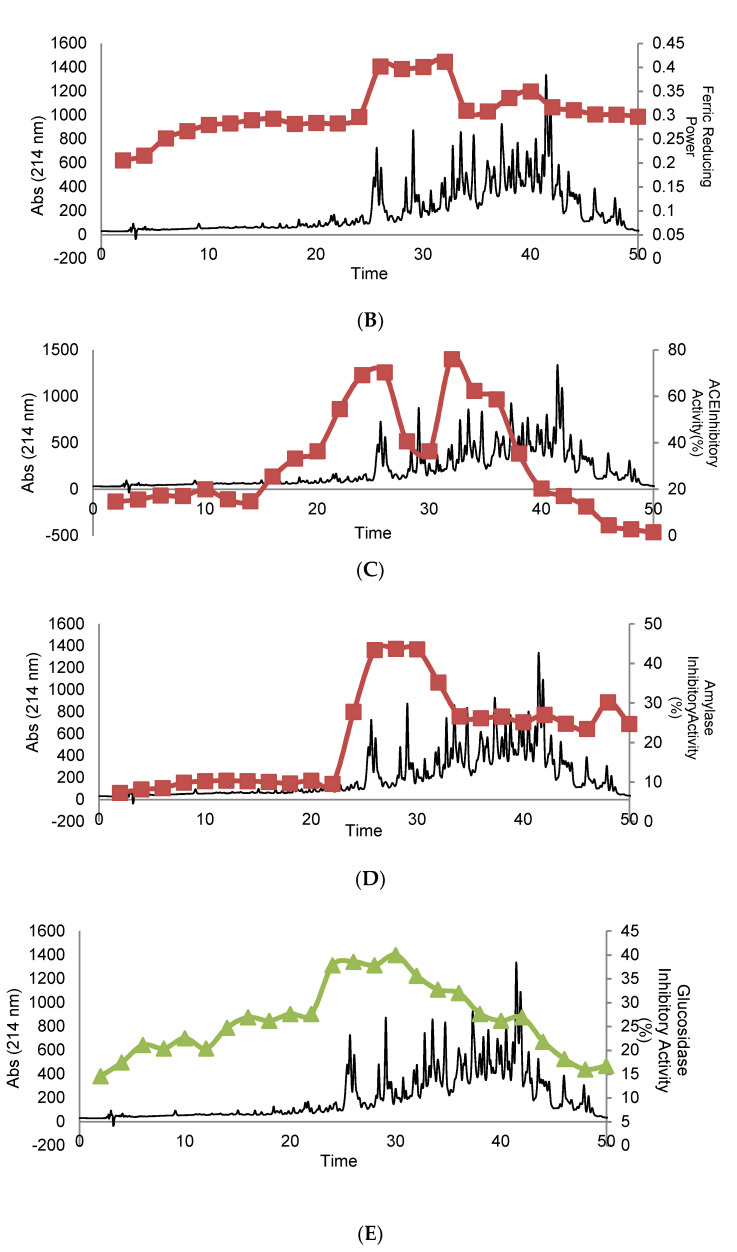
Reversed-phase chromatographic separation of the peptide fractions from 44 to 54. The fractions were automatically collected and assayed for their DPPH scavenging activity (**A**), ferric reducing capacity (**B**), ACEI-inhibitory activity (**C**), α-amylase inhibitory activity (**D**), and α-glucosidase inhibitory activity (**E**).

The biological activity tests of hydrolyzed orange seed peptide fractions of 44–49 and 50–54, purified and isolated using RP-HPLC, and including DPPH scavenging activity, ferric reducing power, ACEI-inhibitory activity, α-amylase inhibitory activity, and α-glucosidase inhibitory activity are illustrated in Figure 6. The results of antioxidant activity tests showed that fraction 32 had the highest DPPH scavenging activity (41.23%) and ferric reducing activity (absorbance of 0.412). The evidence showed that with increasing hydrophobicity, the antioxidant activity also increases. In relation to the ACEI-inhibitory activity, fraction 32 showed the highest value with 75.96%. Based on the chromatogram, peptides with high hydrophilicity and hydrophobicity features demonstrated the highest ACEI-inhibitory activity. The antidiabetic potential tests revealed that fraction 28 had the highest α-amylase inhibitory activity with 43.7% and fraction 30 the highest α-glucosidase inhibitory activity with 40.0%. Finally, fractions 31 and 32 of RP-HPLC were selected for identification due to their biological activity values that showed good results in all the assays.

### 3.5. Effect of In Vitro GI Digestion on the Peptides’ Bioactivity after RP-HPLC Separation

**Figure 7:**
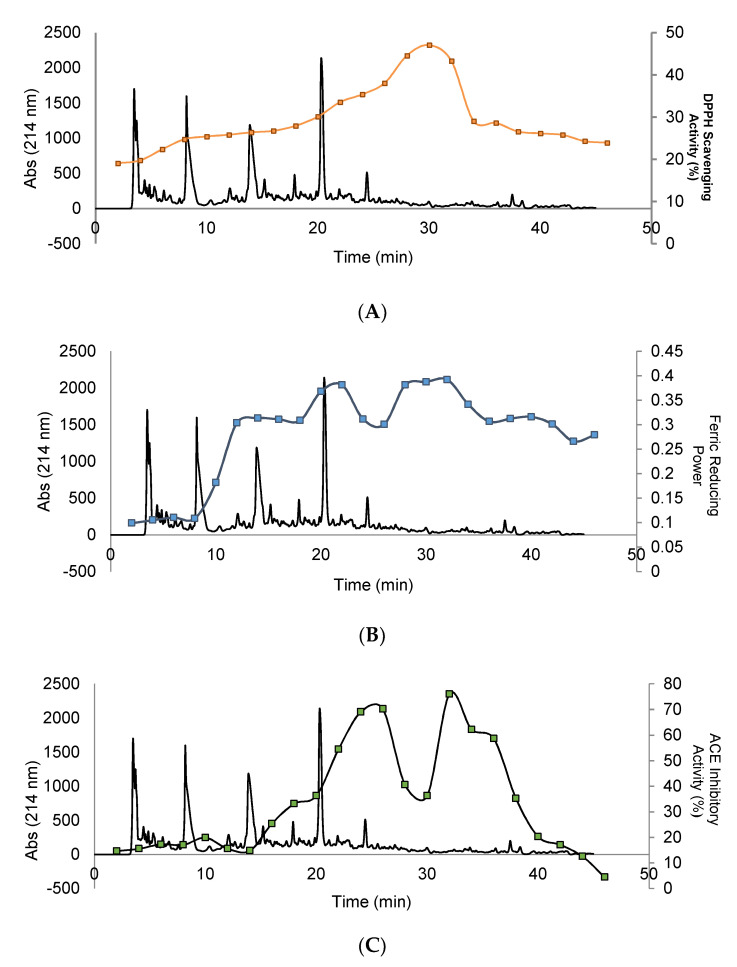

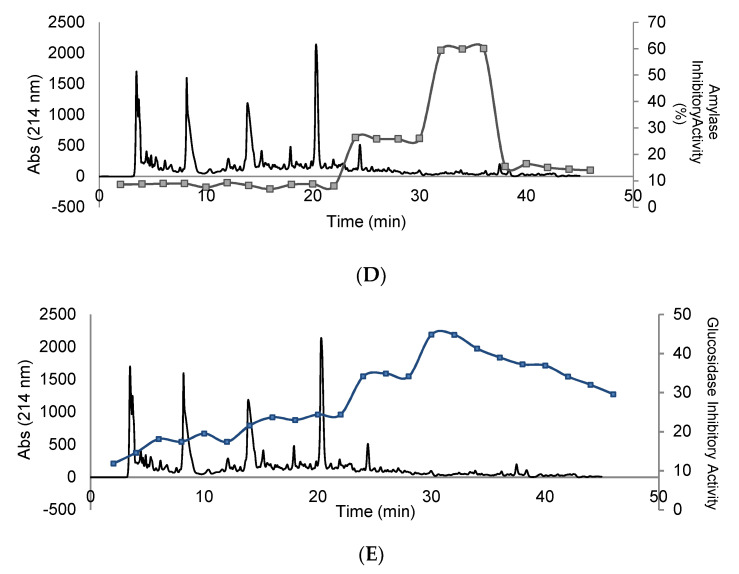
Reversed-phase chromatographic separation after *in vitro* gastrointestinal digestion of the fractions from 44 to 54 obtained after SEC separation. The fractions were automatically collected and assayed for their DPPH scavenging activity (**A**), ferric reducing capacity (**B**), ACEI-inhibitory activity (**C**), α-amylase inhibitory activity (**D**), and α-glucosidase inhibitory activity (**E**).

After in vitro GI digestion, fractions 45–49 and 50–54 were analyzed using RP-HPLC, and their biological activity was evaluated (see Figure 7). The results showed important differences in the chromatographic profile after gastrointestinal digestion in comparison with Figure 6. However, similar bioactive results were obtained. These results showed that the pepsin-hydrolyzed orange seed proteins were highly resistant to simulated gastrointestinal digestion conditions, and their biological activity increased or remained unchanged in all assays except ferric reducing capacity. Therefore, the potential of peptide fractions 31–32 to be used as ingredient in foods should be considered in further studies due to their capacity to resist in vitro gastrointestinal digestion.

### 3.6. Identification of Peptides by Nano-LC-MS/MS

In order to identify and to determine the properties of the peptides, the peptide fractions 31–32 obtained from the last stage of purification (RP-HPLC), were analyzed using nano-LC-MS/MS. In the present study, a total of 952 peptides from hydrolyzed orange seed proteins were identified using Mascot Distiller software, and the results are shown in Table S1 of the Supplementary Material. In Table 1, a total of 26 peptides identified after in vitro gastrointestinal digestion are shown. In this sense, Table S2 of the Supplementary Material also shows the complete data of the identified peptides after gastrointestinal digestion. These tables describe the sequence of amino acids, the mass of the fragment observed in the detector after ionization, the expected mass according to the observed mass, and the ion charge status. Finally, the main amino acids present in the identified peptides were histidine, proline, serine, aspartic acid, glutamic acid, and tyrosine.

## 4. Discussion

Results showed that by increasing the hydrolysis time and the enzyme to substrate ratio, the inhibitory activity of ACEI in the hydrolyzed samples increased. Furthermore, using an enzyme concentration of 0.03 and hydrolysis time of 3.5 h, the antioxidant activity and the inhibitory activity of α-amylase and α-glucosidase enzymes increased. These results are in agreement with previous studies of the antioxidant activity of hydrolyzed peanut proteins and tuna liver proteins, where similarly, by increasing hydrolysis time and concentration, an increase in the antioxidant activity was observed [27,28].

In another study, the highest ACEI-inhibitory activity of 83.70% was obtained using an enzyme to substrate ratio of 0.03 and hydrolysis time of 5 h. In general, increasing hydrolysis time leads to the production of peptides smaller than 3 kDa, which could show higher ACEI-inhibitory activity [29]. Je et al. [28] reported an ACEI-inhibitory activity in fish liver protein of 36% after hydrolysis using various enzymes such as Flavourzyme, Neutrase, Alcalase, and Protamex, which is lower than the activity observed in the present study (83.70%), probably due to the differences in matrices and types of protein. Possible mechanisms of action of peptides showing ACEI-inhibitory activity are (i) the peptides bind to the active site of the ACEI enzyme, or (ii) they may bind to the inhibitory sites located on the ACEI enzyme. These binding alter the protein’s structure and prevent the binding of substrate (angiotensin) to the active site of the enzyme [30]. Moreover, the highest ACEI inhibition was associated with peptides with high hydrophobicity, because hydrophobic peptides show affinity to the active site of the ACEI enzyme [31]. Furthermore, differences in the ACEI-inhibitory activity between treatments were described as differences in the molecular weight and the sequence of amino acids in the peptides [32].

In this study, the highest antidiabetic potential was observed using an enzyme to substrate ratio of 0.03 with a hydrolysis time of 3.5 h. The results indicated that the highest inhibitory activity of α-amylase and α-glucosidase were 42.3% and 45.4%, respectively. Previous studies showed that the content in aromatic amino acids including phenylalanine, tryptophan, and tyrosine in the peptide chain are key factors in the ability to inhibit α-glucosidase or α-amylase enzymes [33,34]. Therefore, the high anti-diabetic capacity of hydrolyzed orange seed proteins could be due to the presence of aromatic amino acids in their structure.

In order to identify the main peptides responsible for the antioxidant, antihypertensive, and antidiabetic activities, sample T6 (0.03 E/S, 3.5 h) was analyzed using SEC. Regarding the observed results, Khantaphant et al. reported that differences in the molecular weight of the peptides’ profile may be related to differences in enzymes’ specific breakdown sites [35]. Thus, the active site of the pepsin enzyme contains thiol groups and breaks the bonds adjacent to the tryptophan, tyrosine, and phenylalanine amino acids [35].

SEC has been used as a common procedure for the peptides’ isolation before RP-HPLC [36,37]. The highest antioxidant activity in the peptide fractions isolated from SEC was in fractions with molecular weights lower than 13,000 Da, and the maximum inhibitory activity of ACEI enzyme was related to peptide fractions with molecular weight less than 13,000 Da. Other studies also implied that low-molecular-weight peptides show higher ACEI-inhibitory activity [25,32,38]. In addition, it has been reported that peptides with high antidiabetic properties contain high amounts of hydrophobic amino acids [39], and they may inhibit α-amylase and α-glucosidase enzymes by binding to the active site of the enzyme through hydrophobic bonds [33]. The affinity of binding to the enzyme active site was enhanced by increasing the peptide bond breakdown points in a protein chain and producing shorter peptide chains [30]. The results showed that by increasing hydrophobicity, the amount of antioxidant activity also increased. Andersen et al. [40] suggested that the most powerful antioxidant compounds usually are strong reducing agents. This is probably due to the high amount of aromatic and hydrophobic amino acids in these peptide fractions. Several researchers documented that the antioxidant properties, ACEI-inhibitory activity, and antidiabetic potential have been closely associated with the ratio of hydrophilic to hydrophobic peptides [11,25,36,37,41].

During the separation of peptides using RP-HPLC, peptide fractions that were more retained in the C18 column showed the highest DPPH activity [42]. The identification of the peptides in this fractions confirmed the presence in the sequence of hydrophobic amino acids such as leucine, phenylalanine, valine, and tryptophan [42]. Power et al. [42] revealed that active antioxidant peptides mainly contain hydrophobic amino acids in their structure. Tryptophan also plays an important role in the inhibitory activity of DPPH through its hydrogenating role [11,17,42]. These results are similar to the results obtained after the hydrolysis of royal jelly [11] and tomato seeds [17]. In relation to the inhibitory activity of the ACEI enzyme, fraction 32 showed the highest inhibitory activity in the obtained peptides. It has been suggested that polar groups present in hydrophilic peptides and non-polar or aromatic groups in hydrophobic peptides may bind to the active site or to the inhibitory site in ACEI enzyme. These bindings would alter the spatial structure of the enzyme, preventing angiotensin from binding to the active site of ACEI [31,43,44]. On the other hand, peptides with high hydrophilicity have less access to the ACEI active site [24,31,32]. Meanwhile, the peptides of the primary chromatogram fractions appeared to be multifunctional peptides, and the reason for this can be related to the balanced content of polar groups of hydrophilic amino acids in the primary fractions [11,30]. The fractions 28 and 30 showed the maximum α-amylase and α-glucosidase enzyme inhibitory activities, respectively. Our outcomes indicated that there was a direct relationship between the hydrophobic content of bioactive peptides and their biological activity. The results of this study were consistent with the results obtained from the biological activity of bioactive peptides from hydrolysis of royal jelly [11] and tomato seeds [17].

In the current study, the bioavailability of peptide fractions was evaluated by simulated gastrointestinal digestion. The stability of the biological activity of peptides depends not only on the maintenance of their structure but also on other factors [45]. Peptide behavior in simulated gastrointestinal digestion may provide important information about their possible behavior in the digestive system. The changes in the peptides profile observed after HPLC separation indicated the important effect of gastrointestinal enzymes on the sequences. Moreover, acidic conditions with pH 2 and increasing to pH 7 could cause changes in peptide sequences. Marambe et al. (2011) demonstrated that acidic treatment reduced some peaks of high-molecular-weight peptides in the chromatogram and increased other peptides of 1 kDa and less [46]. Moreover, Meshginfar et al. reported that the neutral polar charge of ionizable amino acid group in the sequence of peptides was very effective on the stability of peptides in digestive conditions [17]. They also indicated that digestion in antioxidant peptides causes minimal changes in their biological activity [17]. Thus, it seems that the hydrolyzed orange seed protein peptide fractions may retain their biological activity after the gastrointestinal digestion stage.

The fraction 31–32 obtained from the last purification step (RP-HPLC) was applied for the identification of the peptides using nano-LC-MS/MS. A total of 952 sequences were identified in samples before gastrointestinal digestion in comparison with 26 peptide fractions that were identified after gastrointestinal digestion. This decrease in the amount of peptide sequences identified could be due to the intense hydrolysis occurring during gastrointestinal digestion that results in the generation of very small peptides that are out of the analysis range in the mass spectrometer. Selma et al. (2018) suggested that the highest antidiabetic and antihypertensive activity of peptides detected from protein hydrolysates from *Octopus vulgaris* by nano-LC-MS/MS after isolation with RP-HPLC were related to fractions with a molecular mass of about 400–2500 Da and mainly contain the hydrophobic amino acids [39]. Another study on the antioxidant activity of bioactive peptides from the porcine plasma protein hydrolysis concluded that the DPPH scavenging activity and Fe^3+^ ion reduction power were the highest in molecules that weigh less than 3 kDa [47]. Torkova et al. (2016) [48] studied the hydrolyzed peptides of poultry protein and the effect of in vitro gastrointestinal digestion conditions on the bioactive activity of these peptides and showed that all peptides inhibiting the ACEI enzyme had 5 to 9 amino acids in their peptide chain [48]. Our outcomes were consistent with the results of previous studies including Maqsoudlou et al., Meshginfar et al., and Mazloomi et al. [11,17,49], and it was revealed that histidine, proline, serine, aspartic acid, and glutamic acid were the important amino acids in peptides with antioxidant properties. Histidine, proline, serine, glutamic acid, and tyrosine were also the important amino acids in peptides with ACEI-inhibitory properties. Ultimately, proline, serine, aspartic acid, and glutamic acid were also important amino acids in peptides with antidiabetic potential. Generally, the restriction on the number of detected peptides in the hydrolyzed pepsin and the presence of specific amino acids at the end of these peptide chains has been due to the presence of specific amino acids in the active site of the pepsin enzyme [28,35]. This was evident in the amino acid sequence of the detected pepsin-hydrolyzed peptides. Depending on their specific structure, some bioactive peptides can resist the protease enzymes’ action. For example, peptides containing proline at the end of the carboxyl group are more resistant to gastrointestinal digestion and are, therefore, not hydrolyzed by digestive enzymes [11]. On the other hand, due to their molecular weight, and hydrophobicity, some antihypertensive peptides are structurally resistant when passing through intestinal epithelial cells as well as digestive digestion [11]. Mazloomi et al. (2020) [49] showed that orange seeds can be used to produce beneficial bioactive peptides that are resistant to digestive enzymes, and Alcalase-hydrolyzed orange seed proteins can be also suggested as a health-beneficial product to reduce blood pressure and for diabetes management. The study on the peptide sequences after gastrointestinal digestion shows that these peptides may retain their biological activity in many cases and in some cases become new peptides with new health-beneficial characteristics.

## 5. Conclusions

The main objective of this study was to evaluate the antioxidant, ACEI-inhibitory, and antidiabetic activities together with the study of peptides’ stability after in vitro gastrointestinal digestion. The results revealed that the biological activity of pepsin-hydrolyzed orange seed proteins was resistant to gastrointestinal digestion. Thus, pepsin hydrolyzed orange seed proteins could be used as a health-promoting ingredient to help in the reduction of blood pressure and the regulation of diabetes, although future clinical studies in vivo would be necessary to confirm the health beneficial effect of pepsin orange seed protein hydrolysate.

## Figures and Tables

**Table 1:** Peptide sequences identified in the HPLC fractions 31–32 using nano-LC-MS/MS (after gastrointestinal digestion).

Sequence	Amphipathicity	Hydrophilicity	MW	Probable Allergenicity
IQLSAEKGNLYQNA	0.53	−0.11	1548.93	NO
LVDVGNSDNQ	0.12	0.21	1060.23	YES
RVESEAGVTEF	0.57	0.54	1223.45	YES
YQGSQGGEGGDRS	0.48	0.59	1297.45	NO
YQGSQGGEGGD	0.34	0.4	1054.16	YES
QKVESEAGVTEF	0.73	0.51	1323.59	NO
SAEKGNLYPNAL	0.41	−0.02	1276.58	NO
VDVGNSQNQLDQY	0.29	0.02	1479.72	NO
GIETVGGVMT	0.13	−0.39	963.27	YES
GIETVGGVMTK	0.45	−0.08	1091.46	NO
STASDNQNTVTIQV	0.18	−0.15	1477.75	YES
GVNENEYKPEL	0.68	0.62	1291.54	YES
VNENEYKPEL	0.75	0.68	1234.47	YES
FSDEGFGPAPK	0.45	0.35	1151.38	NO
SFSDEGFGPAPK	0.41	0.34	1238.47	YES
FSQPDGPIMGN	0.11	−0.17	1162.44	NO
VVDSIDQDEL	0.25	0.59	1132.33	NO
EGPSLLPDDPEK	0.52	0.97	1296.56	NO
HDLEIGPGAPT	0.25	0.09	1106.36	NO
EGMGGGGGAHDPF	0.21	0.09	1188.43	YES
VDSFHPGEPI	0.27	0	1097.33	NO
DFEDSHPDLPS	0.25	0.71	1258.4	NO
ISVGDKLPDAT	0.33	0.3	1115.4	YES
VFPAGPGGAA	0	−0.55	843.08	YES
VIDANGNLVPH	0.13	−0.38	1148.45	YES
DADGNGTIDFPE	0.11	0.58	1250.4	NO

## Data Availability

The data are contained within the article. The data presented in this study are available in the present article.

## Supplementary Materials

The following are available online at https://www.mdpi.com/2304-8158/10/3/679/s1, Table S1: Complete list of peptide sequences identified in RP-HPLC fractions 31–32 of the pepsin hydrolysate before GI digestion, Table S2: Complete list of peptide sequences identified in RP-HPLC fractions 31–32 of the pepsin hydrolysate after GI digestion.
